# Dexmedetomidine augments the effect of lidocaine: power spectrum and nerve conduction velocity distribution study

**DOI:** 10.1186/s12871-015-0009-9

**Published:** 2015-03-06

**Authors:** Nizamettin Dalkilic, Seckin Tuncer, Ilksen Burat

**Affiliations:** N.E. University, Meram School of Medicine, Biophysics Department, Konya, Turkey

**Keywords:** Neural conduction blockage, Conduction velocity distribution, Power spectrum, Dexmedetomidine, Lidocaine

## Abstract

**Background:**

In this study, the individual and combined inhibitory effects of dexmedetomidine and lidocaine on the conduction group of isolated nerve were investigated by determining conduction velocity distribution (CVD) and power spectrum.

**Methods:**

Electrophysiological compound action potential (CAP) recordings were conducted on isolated rat sciatic nerve before (Con) and 20 minutes after exposure to 1 mM lidocaine (Lido), 21pM dexmedetomidine (Dex) and their combination (Lido + Dex). Then for CVD, mathematical model and for power spectrum Fast Fourier analysis were conducted.

**Results:**

Dexmedetomidine alone made no significant difference in shape and duration of CAPs as compared to Con, on the other hand lidocaine depresses amplitude and prolongs the duration of CAPs, but not more than combination of dexmedetomidine and lidocaine can do.

Lidocaine caused a shift in the CVD histogram to relatively slower conducting group significantly while dexmedetomidine did not cause any significant change as compared to Control. Lidocaine, when combined with dexmedetomidine revealed a remarkable effect on the whole CVD histogram by causing almost complete blockage of fast conducting nerve fibers. The relative number of fibers in CVD is conserved for separate applications of anesthetics, but not for their combination.

As in CVD, power spectrum shifted from higher to lower frequency region by lidocaine and significantly for lidocaine combined with dexmedetomidine application. Shifts for dexmedetomidine applied group were seen beggarly.

**Conclusions:**

We have concluded that dexmedetomidine alone did not influence nerve conduction, but when it is used with lidocaine it augments neural conduction blockage effect, especially on fast conducting nerve fibers.

## Background

Investigation on molecular mechanism by which local anesthetics alter specific function of peripheral nerve system implicates the sodium (Na^+^) channels. It can also affect potassium (K^+^) and calcium (Ca^2+^) channels [1]. Lidocaine (a crystalline compound C_14_H_22_N_2_O) is a local anesthetic; it is used to produce infiltration anesthesia and various nerve blocks. Lidocaine is also believed to inhibit nerve conduction (so propagation of action potential) by blocking sodium ion flux across nerve membrane. This creates the anesthetic effect by not merely preventing pain signals from propagating to the brain but by stopping them before they begin [2].

Dexmedetomidine (C_13_H_16_N_2_) as a specific and selective α2-adrenoceptor agonists is a novel sedative-analgesic agent. By binding to the presynaptic α2-adrenoceptors, it inhibits the release of norepinephrine, terminate the propagation of pain signals [3]. It is also used for anesthetic-sparing effects and analgesia [4]. At high concentrations, it can also affect transcarinal motor evoked responses, [5,6] yet there are limited reports regarding its ionic mechanism of action on neurons.

Information is transferred within the neurons by action potentials with different conduction velocity (CV). The conduction velocity of nerve fibers varies linearly with the diameter of fibers [7]. CV is also closely related with the density of Na^+^ channel in the membrane patch. The two passive membrane parameters; time constant (τ = R_m_C_m_) that is designated by membrane capacity (C_m_) and membrane resistance (R_m_), and the space constant ($\lambda =\sqrt{R_m/{R}_o}$, R_o_: extracellular resistance) are other factors that determine CVs [8]. Consequently the evaluation of the distribution of nerve fiber conduction velocities (CVD) is important for the measurement of nerve function. Using an appropriate CVD estimation model, the functional state of a given fiber group may be monitored before and after a certain event. It was applied so that quantitative comparison between the groups is possible [9-11].

It is pointed out about the sedative effect of dexmedetomidine in the literature but not on neural conduction blockage. Blockage effect of lidocaine is known on neural conduction, yet there is no study on its effect on CVD. This study aims to investigate the comparative inhibitory effect of dexmedetomidine and lidocaine on conduction velocity group of isolated nerve by determining CVD and power spectrum.

## Methods

All animals were cared for in accordance with the National Institute of Health's Guide for the care and use of laboratory animals. Sprague Dawley rats weighing 250-300 g (12-14 weeks old) were used for study. After birth, five rats were housed per cage at ambient temperature and humidity on 12/12 h light/dark cycle. All animals received food and water ad libitum. All chemicals were purchased from Sigma and Merck. The protocol that is used in this study is approved by the Ethical Committee of NE University Experimental Medicine Research and Application Center (Approval number: 2013-186) and experiments were conducted in Meram Medical Faculty Biophysics Research Laboratory.

### Experimental procedure

Under light anesthesia (100 mg/kg Ketamin-HCl (Ketalar®, Pfizer) and 5 mg/kg Ksilazin-HCl (Rompun®, Bayer), i.p.) sciatic nerves were dissected from hind limb of rats killed by cervical dislocation; then transferred into the organ bath, which was perfused with modified Krebs solution (119 mM NaCl, 4.8 mM KCl, 1.8 mM CaCl_2_,1.2 mM MgSO4, 1.2 mM KH_2_PO_4_, 20 mM NaHCO_3_,and 10 mM glucose, having a pH of 7.4 and gassed with a mixture of 95% O_2_ and 5% CO_2_) at a constant rate of 5 ml/min at fixed temperature (37 ± 0.5 °C). Only sciatic nerves from one side of an animal were used for experiments.

Electrophysiological recording experiments were then achieved. Three different exposure procedures were used. For the exposure experiments, recording chamber media was changed with modified Krebs solution that also has a concentration of anesthetics; 1 mM Lidocaine (Xylocaine, AstraZeneca, Sweden) for Lido group, 5 μg/L Dexmedetomidine (Precedex, Hospira Inc., USA) for Dex group, 21 pM Dexmedetomidine combined with 1 mM Lidocaine for Lido + Dex group. In Control group (Con) none of anesthetics were added to the recording chamber. Sciatic nerves were exposed to each anesthetic or anesthetic combination for 20 minutes which is the time required for maximal decrease in CAP amplitude and recording was performed at the 20^th^ minute of the exposure. Throughout the experiments, a sciatic nerve was used only for one group or condition.

For the stimulations square–shaped supramaximal pulses of 0.2 ms duration at a frequency of 1 Hz were given from proximal ends of the nerve trunk through a stimulus isolation unit (Model SIU5; Grass Instruments Co.) using a stimulator (Model Grass S88K). In order to guarantee the recording from the same number of fibers activated at any point along the nerve, CAP recordings were performed from the tibial branch of the isolated nerve trunk by using a suction electrode mounted in organ bath [10,12]. Supramaximal pulses were determined as the stimuli of intensity approximately 20% greater voltages than the required for getting maximal CAP amplitude. Amplified (x1000) and filtered (1Hz to 10 KHz) (CP511 AC Amplifier, Grass Instruments) CAP signals were digitized by 12 bit A/D converter (Advantech PCL-1710LG) with 50 kHz sampling rate and acquired with the BiosigW data acquisition software and stored on a hard disk for further analysis. Signal averaging was not needed due to pure CAP signals.

### Analysing procedure

To investigate the status of neural function before and after lidocaine, dexmedetomidine and lidocaine + dexmedetomidine treatment advanced mathematical procedures were conducted on all CAP recordings.

Conduction velocity of nerve fibers varies linearly with the diameter of the fibers. Evaluation of the CVD is important both for clinical and basic research. A suitable mathematical model that is the non-invasive method is needed to estimate CVDs from CAPs recorded at certain distances from the stimulus site [5,7,13,14]. To obtain the individual nerve conduction group activity we estimate nerve conduction velocity histogram by using the model that we enhanced beforehand [12,15]. Our model based on the model proposed by Cummins et al. [16,17] and it is sensitive mostly to myelinated fibers as it is in other model studies [13,18].

The procedure we have followed for the estimation of CVD depends on the model involving a priori knowledge about single fiber action potential (SFAP) and one CAP recording, [17] then fitting the forward model to experimental CAP data solves the inverse problem and gives the fiber diameter distribution (FDD) so the CVD [19].

It is assumed that a recorded CAP waveform is the result of the superposition of SFAP signals. The nerve fibers can be separated into N classes so that the fibers in each class have the same SFAP waveform. The sampled CAP(t) signal then can be expressed as

1 $$ CAP(t)={\displaystyle \sum_{i=1}^N{w}_i{f}_i\left(t-{\tau}_i\right)} $$

where N is the number of fiber classes; *w*_i_ is the amplitude-weighting coefficient for class i; τ_i_ is the propagation delay for fibers in class i [10,16,17]. The SFAP waveform function f_i_(t) for class i is defined as

2 $$ f\left(t,v\right)=A(v){f}_0\left({v}^q,t\right) $$

where A(v) denotes the amplitude of SFAP with velocity v, f_0_(t) is a fixed “standard” SFAP, q is a constant. Many authors have adopted an empirical relationship related with dependence of SFAP waveform on propagation velocity of the form

3 $$ A(v)={d}^p $$

where d is the fiber diameter and p is a constant [20]. The weighting coefficients (w_i_) are general parameters to account for all influences on the contribution of each fiber class to the observed CAP. Thus, w_i_ may be expressed as

4 $$ {w}_i=A\left({v}_i\right)\cdot H\left({m}_i\right) $$

where m_i_ is the number of fibers activated in class i; A(v_i_) is the dependence of the SFAP amplitude on the conduction velocity; H(m_i_) is the functional dependence of the weighting coefficients on the number of fibers in i [16,17].

To estimate CVD or FDD from recorded CAPs, the problem could be solved by an inverse mathematical procedure to find weighting coefficients in terms of recorded CAPs.

In order to estimate the individual fiber activity from CAPs, CVD for nerves treated with each anesthetic and their combinations were obtained. For the sake of visually augmented effect of dexmedetomidine, lidocaine and lidocaine + dexmedetomidine combination regarding the difference and ease of interpretation three conduction velocity groups were defined intentionally, by dividing CVD into subgroups. The CV subgroups are as, SLOW: 8-28 m/s; MEDIUM: 29-52 m/s; FAST: 53-79 m/s.

The other mathematical procedure is sketch of the power spectrum of the recorded CAPs. To do this Fast Fourier Transform (FFT) procedure was conducted. According to Fourier theorem, any waveform could be constructed by the superposition of a number of independent sine, cosine and constant functions, and hence this waveform consists of different powered signals of different frequencies [21,22]. By using FFT procedure, we have calculated power spectrum of CAPs and the changes in power spectrum with lidocaine, dexmedetomidine and lidocaine + dexmedetomidine were determined and the results were also compared. The problem was whether the spectrum would reveal information about the relative contribution of fiber groups that conduct in different velocities before and after these three drug application.

In spectral analysis, we determined the window time as 5.12 ms. For FFT analysis the number of data points should be power of two, and sampling period is 0.02 ms (sampling frequency is 50 kHz, so it becomes adequate for the Nyquist frequency), 256 data points (2^8^) are enough to comprise from the stimulus artifacts to end of CAP (stimulus artifacts were cleared before FFT analysis). FFT analyzing procedure was conducted on one window for each CAP recording. Since detailed resolution did not give valuable information, frequency axis was divided into 17 bins and each represented various bandwidth given in the graph.

### Statistics

Unless otherwise specified, the comparison of effects for different anesthetics or anesthetic combinations were done with one-way analysis of variance (ANOVA), followed by Duncan post-hoc test for multiple comparisons when analysis of variance indicated significant results. Student’s t-test was used to recognize whether effect of applications is reversible or not. Additionally, p < 0.05 values were taken as significant. Data are presented as mean ± standard error of the mean (SEM) throughout the text.

## Results and discussion

**Figure 1:**
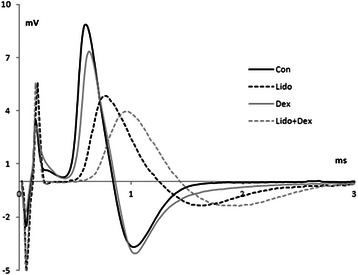
**Sample CAP traces from a single nerve for each groups (Con, Lido, Dex, Lido + Dex) recorded 40 mm away from the stimulating electrodes.**

We have observed that treating the rat sciatic nerve with lidocaine depresses the associated CAPs more as compared with dexmedetomidine. Sample CAP traces are given in Figure 1 for each group in the same time axis. As shown in the figure, dexmedetomidine made no difference in shape of CAP as compared to Con. Lidocaine depresses the CAP (56.00 ± 5.17%), but not as much as compared with lidocaine + dexmedetomidine combination (78.53 ± 3.21%), and this combination also makes latency period the longer. The average amplitudes (mV) of CAPs were 3.93 ± 0.41 for lidocaine, 8.60 ± 0.82 for dexmedetomidine and 1.97 ± 0.37 for lidocaine and dexmedetomidine combination treatments. When compared with Con (9.05 ± 0.50 mV) the average amplitudes were significantly different (p < 0.05) for the groups except Dex. After lidocaine and lidocaine + dexmedetomidine treatments, the nerves were returned to the drug-free solution for 25 minutes (washout) and the average amplitudes were 8.58 ± 0.17 mV and 8,43 ± 0.34 mV, respectively, which means complete recovery of the CAPs (p < 0.05).

**Figure 2:**
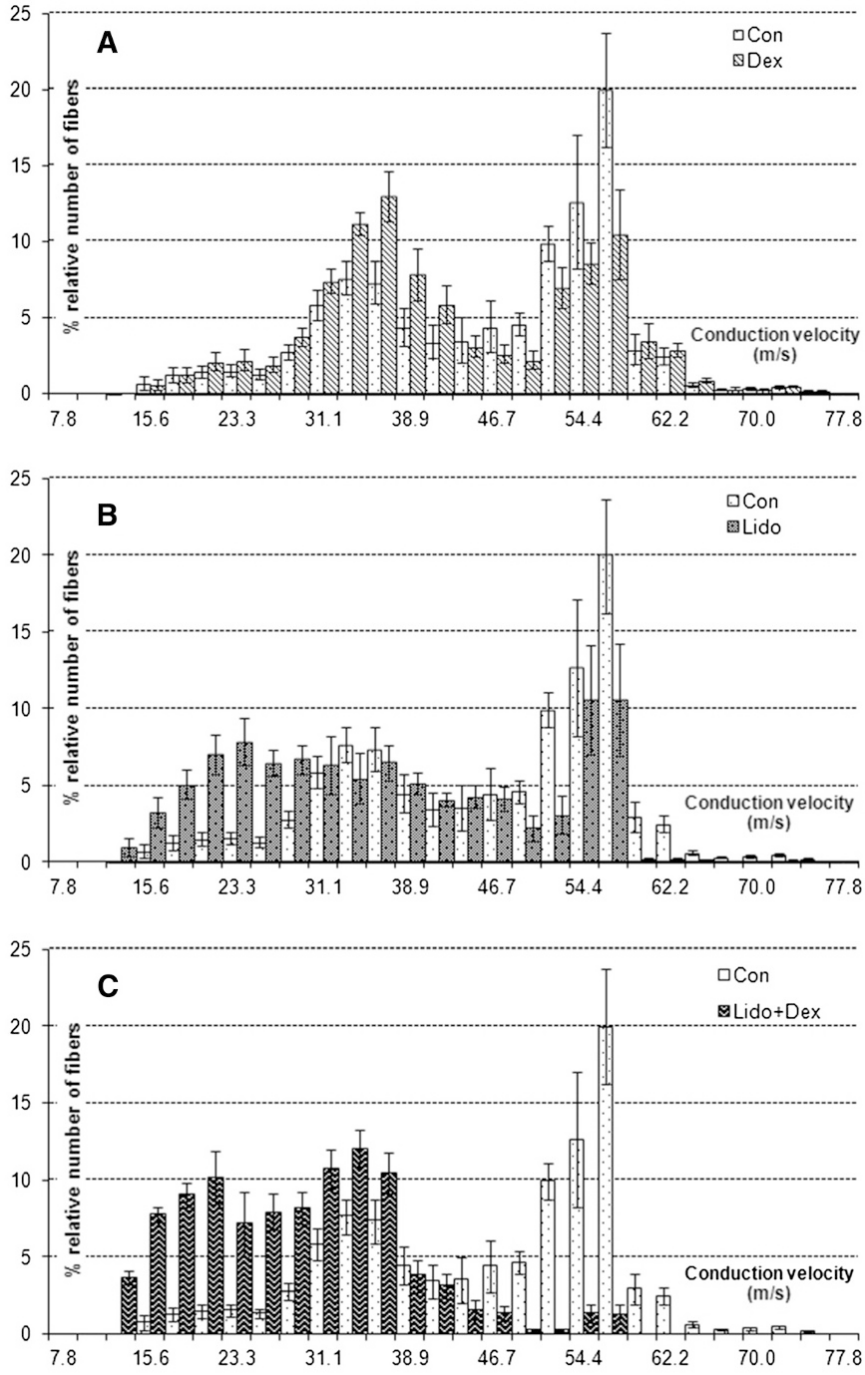
**Estimated separate CVD histograms of Lido, Dex and Lido + Dex groups.** For comparison, in each graph, Con histogram is also given in the same conduction velocity (m/s) axis with Dex **(A)**, Lido **(B)** and Lido+Dex **(C)**. Each bin represents percent relative number of fiber of that conduction velocity class. Values are given as mean ± SEM (N = 7).

The estimated CVD histogram for Con-Dex, Con-Lido and Con-Lido + Dex groups in the same conduction velocity (m/s) axis are given separately in Figure 2A, 2B, 2C. The bins of each conduction groups represent % relative number of fiber of that conduction velocity class. Values are given as mean ± SEM (N = 7).

**Figure 3:**
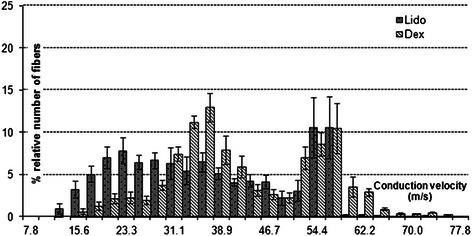
**Estimated CVD histograms of Lido and Dex groups.** Histograms are given in the same conduction velocity (m/s) axis comparatively. Each bin represents percent relative number of fiber of that conduction velocity class. Values are given as mean ± SEM (N = 7).

The comparative CVD histograms of Dex and Lido groups are given in Figure 3 in the same conduction velocity (m/s) axis. Each bin in the axis in the graph also represents % relative number of fiber of that conduction velocity class. Values are given as mean ± SEM (N = 7).

**Figure 4:**
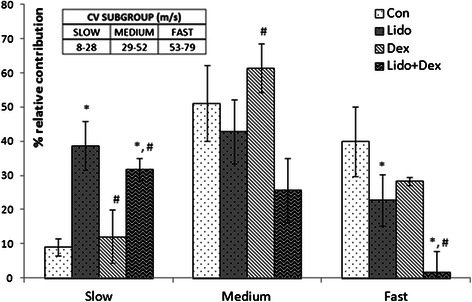
**Recalculated conduction velocity distribution of three subgroups for four experimental group of constituted conduction subgroups as described in the Methods (slow:8-28 m/s; medium:29-52 m/s; fast:53-79 m/s) for the groups Con, Dex, Lido and Lido + Dex.** * represents the significance when compared to the Con group while # represents the significance when compared to the Lido group at a level of p < 0.05.

Intentionally redefined conduction velocity subgroups that range as *slow*: 8-28 m/s; *medium*: 29-52 m/s; *fast*: 53-79 m/s are given in Figure 4 as bar graphs for Con, Lido, Dex and Lido + Dex, so that relative numbers of active fiber were recalculated. Values are given as mean ± SEM (N = 7). Borders of subgroups are defined according to velocity where remarkable changes take place.

**Figure 5:**
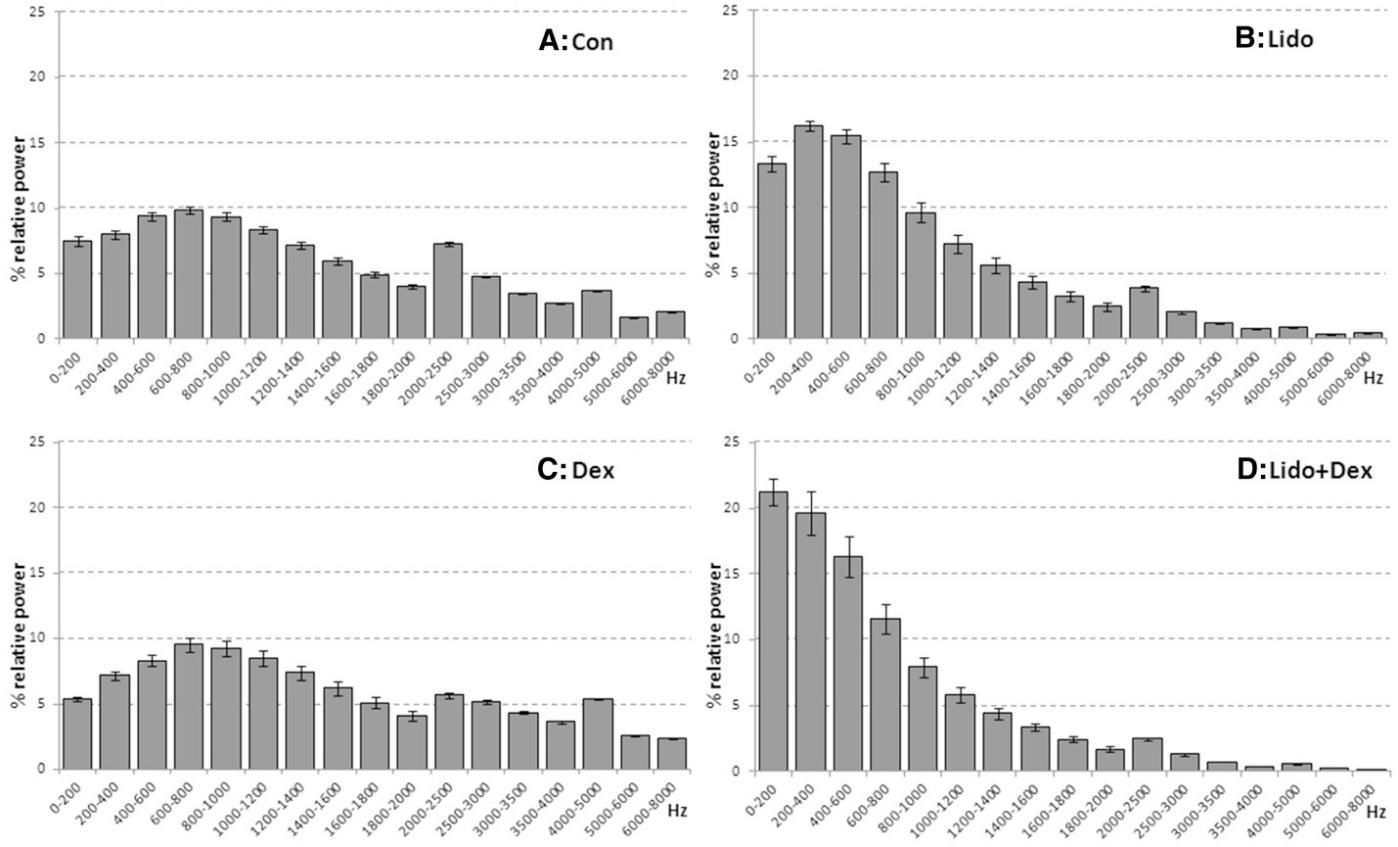
**The effect of lidocaine, dexmedetomidine and lidocaine + dexmedetomidine combination on CAP waveform power spectra.** Graphs show the % relative power vs. frequency relationship related to CAPs for control **(A)** and three different drug groups **(B,C,D)**. Frequency axis is divided into 17 bins.

Relative percentage power values for four experimental groups versus frequency bin graphics are given in Figure 5. All values are given as mean ± SEM (N = 7). In all graphs, frequency bands ranges between 0-8000 Hz. As seen in the graph almost all frequency bands have more or less relative power components in the Con and Dex groups. Yet relative power components of Lido and but especially for Lido + Dex group decrease dramatically as the frequency increases. Relative power of Lido + Dex shifts to lower frequency bands. In relatively high frequency of 3000-8000 Hz bands relative power comprises approximately 14% and 16% for Con and Dex while it is 5% and 2% for Lido and Lido + Dex, respectively. It also comprises in relatively low frequency of 0-800 Hz bands 34% for Con and 28% for Dex while it is 46% for Lido and 56% for Lido + Dex.

The current study was designed to test how the anesthetic agents lidocaine and dexmedetomidine effect neural conduction when they are applied separately and together by using numerical analyzing methods to CAP signals of isolated rat sciatic nerve in organ bath. From the individual CAP signals we have observed that direct application of Dex on isolated nerve has not made any significant change (Figure 1). On the other hand, lidocaine depletes CAPs amplitude, and lengthens the duration and latency period, while dexmedetomidine makes little difference in shape and duration of its CAPs. Nevertheless, lidocaine and dexmedetomidine combination have the most significant effect as compared to those seen in lidocaine and dexmedetomidine treatments alone. Additionally the effect of the tested anesthetics and their combinations on CAP is found to be reversible. From the figure it can also be seen that latency period of CAP for Lido + Dex group is the greatest, meaning that the most decrement in CV is in this group. Although in a study by Kosugi et al. [23], amplitudes of CAP signals recorded from frog sciatic nerves have found to be decreased by application of high concentrations of dexmedetomidine (0.5 mmol/L), in our study the average of the CAP amplitudes did not change significantly by dexmedetomidine alone. This discrepancy can be associated with the dose difference.

Determination of the conduction velocity histogram is a numerical method and when applied to recorded CAP one may gather information on the relative number of active fibers for discrete conduction velocity values in a nerve trunk [10,11,16,17]. Hence with CVD the functional state of a given fiber group may be monitored before and after a certain event, so that making quantitative comparison between groups would be possible. In this study our problem was if CVD would reveal information about the relative contribution of fibers groups before and after lidocaine, dexmedetomidine and lidocaine + dexmedetomidine treatment.

Our findings from the Figure 2 suggest that the CVD bins as compared with those for Con tend to shift to left (to slow the conduction velocity classes) for Lido, Dex and Lido + Dex groups. Little shift in the CVD bins for Dex group is seen but it is not so significant (Figure 2A). This means application of dexmedetomidine alone has not so much effect on neural conduction. So, it can be deduced from this result that, Dexmedetomidine as a selective α_2_-adrenergic receptor agonist has little or any potency on membrane channel when applied on axon as a single as well as on passive membrane properties (on λ and τ).

Application of lidocaine makes respectable shift in the CVD bins to left and reduction in relative number of fibers (%) as compared with Con group. This implies that lidocaine when applied alone has noteworthy impact on neural conduction (Figure 2B). Comparison of the CVD for Lido with that for Dex groups also show that the shift in bins to left for Lido are more significant than for Dex (Figure 3). Lidocaine, as a common local anesthetic with sedative, analgesic, and cardiac depressant properties is known to alter signal conduction by blocking the fast voltage gated Na^+^ channel in the neural cell membrane [3]. Though at this concentration level (1 mM) of lidocaine the generation of action potentials slows down, but not complete blockage of conduction is seen, so the total relative number (%) of fibers is conserved.

The combined implementation of lidocaine with dexmedetomidine makes also remarkable impact on neural conduction. Even though the shift to left in the estimated CVD for Lido is much more than that for Dex group (Figure 3), there is significant increase in proportion of relatively slow conduction class, and complete vanish in proportion of relatively fast conduction class and there also may be in other classes for the Lido + Dex (Figure 2C). Additionally unlike Lido group, total relative number (%) of fiber of Lido + Dex is not conserved. From these results we may say dexmedetomidine increases the forcefulness of lidocaine on neural conduction blockage.

Relative numbers of active fibers were recalculated according to newly defined CV subgroups (SLOW, MEDIUM and FAST) for four experimental groups (Figure 4), as the details are given in methods. We noticed that the most contributing fibers groups to CAP are MEDIUM (~50%) and FAST (~40%) in Con, while contributions to SLOW (~10%) are relatively small. Beginning with FAST subgroups, relative number of fibers for Lido (~22%) decreases significantly (p < 0.05) and for Dex (~28%) it also decreases but not significant as compared with Con. In contrast to these it completely vanishes for Lido + Dex group (p < 0.05).

Continuing with the MEDIUM subgroups there are decrement for Lido (42%) while significant increment for Dex (61%, p < 0.05) as compared with Con. The increment means that Dex makes a little bit slow down in the conduction velocity of FAST subgroup, so it shifts to MEDIUM subgroup. There is also significant decline for Lido + Dex (25%, p < 0.05) in this subgroup, meaning that it suppresses the generation of nerve action potentials more than Lido. Lastly for SLOW subgroup, little increment is seen for Dex (~12%) while substantial rise for Lido (40%) and Lido + Dex (35%). These results show that Dex made little lessenings in conduction velocity of all nerve conduction classes. Lidocaine, on the other hand depletes the conduction velocity of all conduction classes significantly (p < 0.05), yet total % relative number of fiber is conserved. Lidocaine and dexmedetomidine combination, conversely, not only diminish the conduction velocity but also make complete block in nerve conduction, specifically for FAST subgroup.

Frequency component of CAP as relative power (%) can be derived by Fast Fourier Transform analysis, [21,22] as the detail is given in method section. Provided that the influence of conduction distance is eliminated, each SFAP determines the amplitude and shape of CAP, so the FFT histogram [20]. The CAP's frequency constituents appears to be in 0-8000 Hz interval for the control group and it is divided in 17 bins, and each bin represents relative power (%) of that frequency (Figure 5). The spectrum for Dex is not so much different from that of Con. The spectrum shifts from higher to lower frequency region for Lido and especially for Lido + Dex. Shifts for Dex are seen at a minimum level. Almost all of the components for frequency above 3000 Hz vanish for Lido + Dex. Above 3000 Hz, relative power comprises 15% and 16% for Con and Dex, respectively, while it is 5% for Lido and less than 2% for Lido + Dex. Unlike high frequency region, there is remarkable rise in relative power of 0-800 Hz bands for Lido and especially for Lido + Dex.

The existence of inverse proportion between single fiber action potential (SFAP) duration and nerve conduction velocity is known [20,24]. So, the SFAP of fast conducting fibers’ duration are smaller, while the that of slowly conducting fibers are relatively longer and hence have lower frequency component. The (%) relative power's shifting from high frequency to lower frequency components show blockage in relatively fast conducting fibers. Hence, this finding also supports our CVD findings.

Since CAP is superposition of single fiber action potential’s (SFAP) (Equation 1), contribution of SFAP to CAP determine amplitude and shape of the CAPs [10,14]. Suppression of one of SFAPs influences the CAP's shape, and hence will result in the shift of relative power. Shifting from high frequency to lower frequency component for Lido + Dex group shows blockage in conduction. From blockage of high frequency component and elongation in latency period shown in Figure 1 (that gives conduction velocity of fastest fibers), it may be inferred that lidocaine and dexmedetomidine combination block firstly the fast conducting fibers. Fast conducting fibers which have larger axon diameter is generally constituted by sensory nerve and local anesthetic’s inclination to block small nerve fibers sooner than larger fibers are also known [25]. Our finding for lidocaine and dexmedetomidine combination seems to be contradictory, but it is consistent with the study which explains action of local anesthetics by blocking the pore of Na^+^ channels by binding to a receptor site [2]. In many studies that testing the effect of dexmedetomidine combined with lidocaine, dexmedetomidine has found to be enhancing the local anesthetic effect of lidocaine via α-2 adrenoceptor by using the method described by Bulbring and Wajda [26,27] and tail-flick latency assessments [28]. Although these studies have revealed the similar results with our study, CVD and power spectrum analysis from *in vitro* CAP recordings provide detailed information about the effect of anesthetics and their combinations on nerve fiber having different conduction velocities.

## Conclusions

In conclusion, detailed information about the individual and combined effects of lidocaine and dexmedetomidine on specific conduction velocity groups are provided in the study. As a comprehensive finding, we may conclude that dexmedetomidine alone has no significant influence on nerve conduction; however, when combined with lidocaine, it significantly augments the neural conduction blockage caused by lidocaine. Furthermore, lidocaine by itself, blocks the fast conducting nerve fiber, shifting the CVD histogram to left (in favor of the slower velocities), which is a property not observable for dexmedetomidine. On the other hand, lidocaine, when combined with dexmedetomidine, manifests a remarkable effect on the whole CVD, causing almost complete conduction blockage of fast conducting nerve fibers. Thus, dexmedetomidine, as a α2-adrenoceptor agonist, is shown to be much more effective when combined with a local anesthetic. Our results can guide to future studies about anesthetic drug interactions, yet the efficient doses to be used in clinical applications still needs further investigation.
